# Antibiotic Residues in Milk and Milk-Based Products Served in Kuwait Hospitals: Multi-Hazard Risk Assessment

**DOI:** 10.3390/antibiotics13111073

**Published:** 2024-11-11

**Authors:** Maha S. Alenezi, Yasmine H. Tartor, Mohammed El-Sherbini, Elena Pet, Mirela Ahmadi, Adel Abdelkhalek

**Affiliations:** 1 Food Safety, Hygiene and Technology Department, Faculty of Veterinary Medicine, Mansoura University, Mansoura 35516, Egypt; maha-sh-alenez@std.mans.edu.eg (M.S.A.); elsh@mans.edu.eg (M.E.-S.); 2 Microbiology Department, Faculty of Veterinary Medicine, Zagazig University, Zagazig 44511, Egypt; 3 Management and Rural Development Department, Faculty of Management and Rural Tourism, University of Life Sciences “King Mihai I”, 300645 Timisoara, Romania; elenapet@usvt.ro; 4 Biotechnology Department, Faculty of Bioengineering of Animal Resources, University of Life Sciences “King Mihai I”, 300645 Timisoara, Romania; 5 Food Safety, Hygiene and Technology Department, Faculty of Veterinary Medicine, Badr University in Cairo (BUC), Badr City 11829, Egypt; adel.abdelkhalek@buc.edu.eg

**Keywords:** antibiotic residues, food safety, Delvotest SP-NT, HPLC, milk, risk, beta-lactams, tetracyclines, safety margin

## Abstract

Antimicrobial resistance (AMR) poses a significant global health challenge affecting food safety and development. Residues of antibiotics in food from animal sources, particularly milk, contribute to the development and spread of AMR, alter intestinal microbiota, and potentially lead to allergies, serious health conditions, and environmental and technological problems within the dairy industry. Therefore, this study investigated the residue levels of veterinary drugs from β-lactam antibiotics and tetracyclines in milk and milk products and assessed human health risks. Two hundred milk and milk product samples (pasteurized milk, sterile milk, soft white cheese, and processed cheese, 50 each) were collected from different hospitals in the State of Kuwait and screened for antibiotic residues using a microbial inhibition assay (Delvotest SP-NT) and high-performance liquid chromatography (HPLC). Delvotest SP-NT and HPLC analyses showed that 30, 28, 26, and 24% of the pasteurized milk, sterilized milk, white soft cheese, and processed cheese samples tested positive for antibiotic residues. Forty-eight milk and cheese samples were confirmed as positive by both methods, and six samples initially found to be negative by Delvotest SP-NT were confirmed as positive by HPLC. Multi-antibiotic residues were detected in five samples by using HPLC. The kappa coefficient (0.921; *p* < 0.0001) revealed complete concordance between the HPLC and Delvotest SP-NT results. Ampicillin was the most abundant residue in the positive samples (31.48%), ranging from 2.44 to 3.89 μg/L, with an overall mean concentration of 3.492 ± 0.094 μg/L, followed by tetracycline and oxytetracycline (27.78% each), ranging from 54.13 to 220.3 μg/L and from 41.55 to 160.7 μg/L, with mean concentrations of 129.477 ± 14.22 and 91.86 ± 9.92 μg/L, respectively. The amoxicillin levels in the samples (12/54; 22.22%) ranged from 3.11 to 5.5 μg/L, with an overall mean concentration of 3.685 ± 0.186 μg/L. The maximum concentrations of ampicillin, amoxicillin, and tetracycline were detected in processed cheese with mean concentrations of 3.89 ± 0.28 µg/L, 3.95 ± 0.15 µg/L, and 170.3 ± 0.27 µg/L, respectively. Pasteurized milk contained the maximum concentrations of oxytetracycline, with a mean concentration of 120.45 ± 0.25 µg/L. The tetracycline residues exceeded the standard maximum residue limits (MRLs; 100 µg/L) in 6% of both pasteurized and sterilized milk samples, and in 4% of processed cheese. Additionally, the oxytetracycline levels in pasteurized milk (6%) and amoxicillin levels in processed cheese (2%) were higher than the permitted MRLs (100 µg/L and 4 µg/L, respectively). Furthermore, the antibiotic residues detected in 12.5% (25/200) of the samples were close to standard permissible MRL limits for ampicillin (5%), amoxicillin and oxytetracycline (3% each), and tetracycline (1.5%). Hazard quotients, which compare the standard acceptable daily intake (ADI) to the estimated daily exposure (EDI), indicated that the overall risk associated with antibiotic residues in these dairy products is low. The EDI was lower than the ADI for the tested antibiotics, indicating an elevated safety margin. While the overall hazard quotients are low, the potential for the development of antibiotic resistance due to long-term exposure to low levels of antibiotics should be considered. Hence, strict regulations and enforcement are necessary to prevent excessive residue levels and to promote responsible antibiotic use in dairy production. Regular monitoring of antibiotic residues in dairy products is essential for ensuring consumer safety.

## 1. Introduction

Antibiotics have long been used to treat and prevent infections and as growth promoters in veterinary medicine. Without strict regulations, antibiotics can also contribute to food contamination through residues that induce serious health conditions, especially the development of antibiotic resistance [1]. Antimicrobial resistance (AMR) is one of the major threats to global health, food safety, and development [2,3]. Antibiotic resistance has become a global health concern because it is attributed to the death of approximately 0.7 million people every year, which is expected to rise to 10 million per year by 2050 if no measures are undertaken [2]. Most deaths occur in Africa and Asia, with over four million deaths anticipated in each region. While the death toll estimates for the rest of the world are lower, in both Latin America and Europe, they might still approach 400,000 [4]. AMR not only causes deaths and disabilities, but also has significant economic consequences. The World Bank predicts that AMR could increase healthcare costs by USD 1 trillion by 2050 and result in gross domestic product (GDP) losses of USD 1 trillion to USD 3.4 trillion per year by 2030 [5]. One of the main concerns about cow milk is that it might be contaminated with antibiotics administered to cows to treat infections like mastitis, endometritis, and respiratory problems [6]. Common antibiotics used in food animals for growth enhancement and the prevention and treatment of infections include sulfamethoxazole, enrofloxacin, benzylpenicillin, tylosin, amoxicillin, trimethoprim, oxytetracycline, ampicillin, amikacin, neomycin, doxycycline, tetracycline, tilmicosin, and colistin sulfate [7].

Overuse, misuse, and failure to complete courses of antibiotics in veterinary medicine are significant contributors to the development of antibiotic-resistant pathogens [8]. Although the use of antibiotics as growth promoters in animals has been restricted, antibiotic residues can contaminate food, especially milk, leading to environmental, technological, and health issues. These residues can contribute to AMR, transfer of resistant bacteria, disruption of the intestinal microbiota, allergies, and serious health conditions such as anaphylactic shock, nephropathy, bone marrow damage, carcinogenic and mutagenic changes, and reproductive issues [9,10,11]. Studies have shown that the effects of antibiotics on humans can be cumulative, suggesting that repeated exposure to antibiotics in the environment may have a lasting effect on the human microbiome across generations [12].

Veterinarians play a crucial role in One Health by connecting humans, animals, and the environment. Their responsibility to combat AMR is significant, and collaboration with human medicine is essential for effective prevention and control [13]. Each country should establish regulations regarding the use of antibiotics in livestock and the permissible residual amounts of these drugs in food [1]. Various tests can be employed to detect antibiotic residues to ensure high-quality standards and protect consumer health. These tests can be conducted voluntarily or as part of official regulations, in accordance with European legislative requirements. Ideally, a test should be capable of identifying a broad spectrum of antibiotic residues, quick to perform, and affordable [14]. While the existing methods for the detection of antibiotic residues differ in terms of accuracy, speed, and cost, they generally fall into four categories: microbiological inhibition, biosensors, immunochemical techniques, and chromatographic methods [15,16,17,18,19].

Food and Drug Administration (FDA) regulations have been instrumental in safeguarding food from animal drug residues, including those that can cause allergies, toxicity, or cancer. Although the FDA may authorize certain new animal drugs, it imposes strict conditions: the drug must be used in minimal amounts, it cannot cause cancer, and there must be no detectable carcinogenic residue in animal products after a specified withdrawal period (WP) [20]. European Regulation (EC) No. 853/2004 stipulates that milk for human consumption must not contain antibiotic residues in quantities greater than the established maximum residue limits (MRL) [21]. The MRL, as established by Commission Regulation (EU) No. 37/2010, represents the maximum acceptable concentration of veterinary drugs in food of animal origin [22]. Regulation (EU) 2019/6 established the WP for antibiotics administered to a food-producing animal based on the MRL value. During this period, animal products are not intended for human consumption [23]. 

To evaluate risks from antibiotic residues, the hazard quotient (HQ) and hazard index (HI) were developed to estimate and control human exposure to multiple chemical substances and their potential health problems [24,25]. The food safety margin (FSM) was created using the HQ and HI principles, but it also considers the uncertainty and variation in factors that affect their calculation [26]. The FSM determines whether the difference between the estimated daily intake (EDI) and the acceptable daily intake (ADI) for food chemicals is large enough [27].

Given the increasing concerns about AMR and food safety, limited research has been conducted on the detection of antibiotic residues in milk and milk-based products in Kuwait hospitals. Moreover, the potential public health risks associated with dietary exposure to antibiotic residues remain unclear. To address this, we investigated, for the first time, the prevalence of antibiotic residues in milk and its products in Kuwait hospitals, compared the results of quantified residues with their respective MRLs, and investigated the associated human health risks.

## 2. Results

### 2.1. Frequency of Antibiotic Residues in Milk and Milk Product Samples 

**Figure 1:**
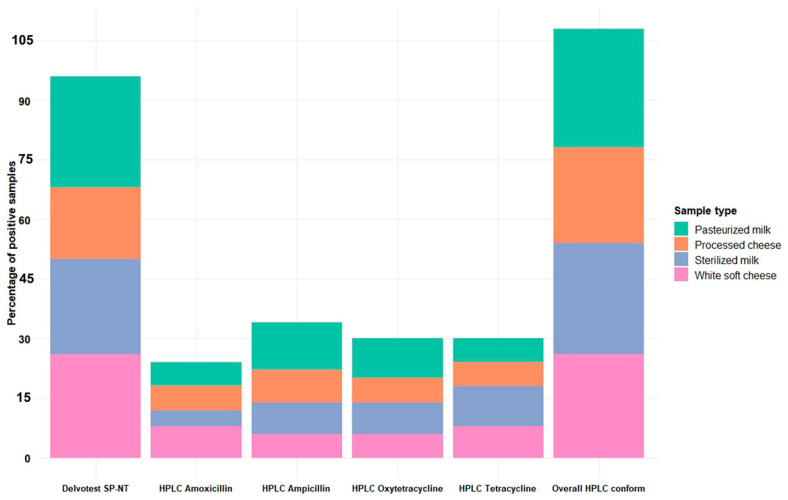
Overall prevalence of different antibiotics residues in milk and milk product samples obtained using Delvotest SP-NT and HPLC.

Overall, the Delvotest SP-NT and high-performance liquid chromatography (HPLC) analyses showed that 30% (15/50), 28% (14/50), 26% (13/50), and 24% (12/50) of the pasteurized milk, sterilized milk, white soft cheese, and processed cheese samples tested positive for antibiotic residues (Figure 1 and Supplementary Table S1). Notably, 73% (146/200) of the samples yielded negative results.

Although the Delvotest SP-NT screening test showed that 24% of the total samples (48/200) were positive, the HPLC analysis revealed that 27% (54/200) of the tested samples were contaminated with β-lactam and tetracycline residues (Table 1). As presented in Figure 1, the highest frequencies of positive results found by Delvotest SP-NT pre-screening were obtained for pasteurized milk (28%), followed by white soft cheese (26%) and sterilized milk (24%).

Forty-eight milk and cheese samples were confirmed as positive by both methods, and six samples initially found negative by Delvotest SP-NT were confirmed as positive by HPLC (Table 1 and Table 2). Furthermore, out of forty-eight samples that were positive by Delvotest SP-NT, multi-antibiotic residues were detected in five samples by HPLC (Table 2 and Supplementary Table S1). Ampicillin and amoxicillin were detected in two types of samples (pasteurized milk and white soft cheese) and both tetracycline and oxytetracycline were found in three types of samples (pasteurized milk, sterilized milk, and processed cheese). 

Figure 1 demonstrates that the frequencies of positive results for ampicillin residue using HPLC in the examined pasteurized milk, sterilized milk, white soft cheese, and processed cheese samples were 12% (6/50), 8% (4/50), 6% (3/50), and 8% (4/50), respectively. The detection rate of amoxicillin was highest in white soft cheese (4/50, 8%), followed by pasteurized milk and processed cheese (3/50, 6% in each), and then by sterilized milk (2/50, 4%). Tetracycline and oxytetracycline residues were detected in 7.5% (15/200) of the examined samples. The highest frequencies of tetracycline residues were in sterilized milk (5/50, 10%), followed by white soft cheese (4/50, 8%), pasteurized milk, and processed cheese (3/50, 6% in each). The frequency of oxytetracycline residues was higher in the milk samples (5/50, 10%, and 4/50, 8%, in pasteurized milk and sterilized milk, respectively) than in the cheese samples (3/50, 6%, in both processed cheese and white soft cheese). Although differences among the samples were observed, they were not statistically significant (*p* > 0.05) (Supplementary Table S1).

Cohen’s kappa revealed a complete concordance between the HPLC and Delvotest SP-NT results when comparing the overall number of findings. The kappa coefficient was 0.921 (*p* < 0.0001), which lies between 0.81 and 1, according to Landis–Koch scale, indicating perfect agreement between Delvotest SP-NT and HPLC (Table 1).

### 2.2. Concentrations of Antibiotic Residues in Milk and Milk Product Samples 

**Figure 2:**
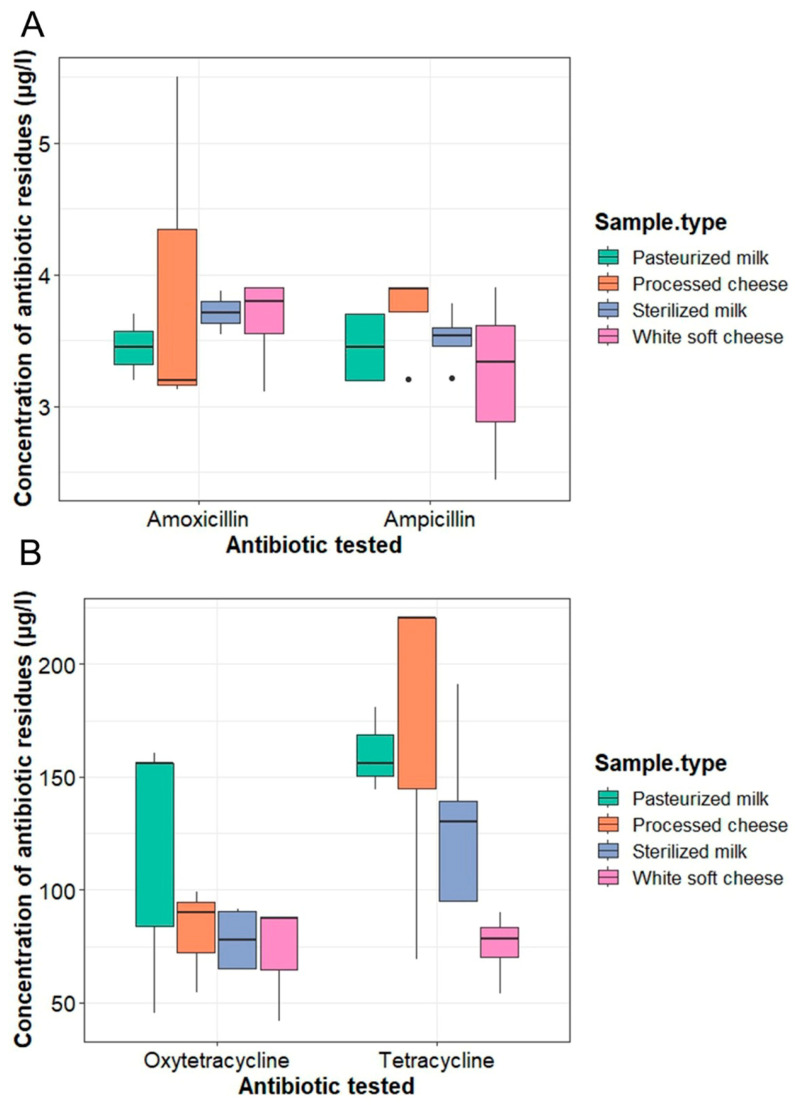
Concentrations of ampicillin, amoxicillin (**A**), tetracycline, and oxytetracycline (**B**) residues in milk and milk product samples using HPLC.

Notably, ampicillin was the most abundant residue in the positive samples (17/54; 31.48%), ranging from 2.44 to 3.89 μg/L, with an overall mean concentration of 3.492 ± 0.094 μg/L, followed by tetracycline and oxytetracycline (15/54; 27.78% each), ranging from 54.13 to 220.3 μg/L and 41.55 to 160.7 μg/L, with mean concentrations of 129.477 ± 14.22 and 91.86 ± 9.92 μg/L, respectively (Figure 2 and Supplementary Table S2). The amoxicillin level in the samples (12/54; 22.22%) ranged from 3.11 to 5.5 μg/L, with an overall mean concentration of 3.685 ± 0.186 μg/L. 

The maximum concentrations of ampicillin, amoxicillin, and tetracycline were detected in processed cheese, with mean concentrations of 3.89 ± 0.28 µg/L, 3.95 ± 0.15, and µg/L, 170.3 ± 0.27 µg/L, respectively. Pasteurized milk contained the maximum concentrations of oxytetracycline, with a mean concentration of 120.45 ± 0.25 µg/L (Figure 2). 

### 2.3. Maximum Residue Limits and Risk of Antibiotic Residues in Milk and Cheese Samples

**Figure 3:**
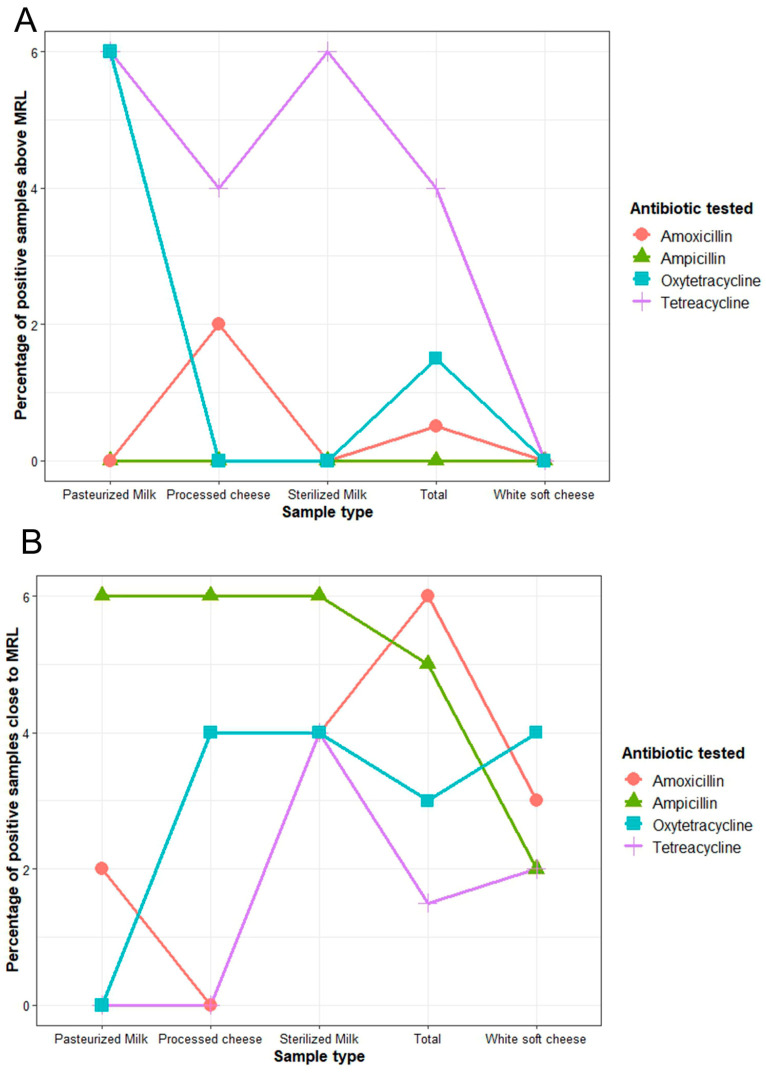
Distribution of examined milk and milk product samples contaminated with antibiotic residues close to the maximum residual limit (MRL) (**A**), and above the MRL (**B**). The values of the MRL for ampicillin and amoxicillin = 4 µg/L each, and tetracycline and oxytetracycline = 100 µg/L each, according to Codex Alimentarius Committee [28]. Close to MRL for ampicillin and amoxicillin > 3.5 µg/L each, and for tetracycline and oxytetracycline > 85 µg/L each.

The results revealed that 6% (12/200) of the tested samples were above the Codex Alimentarius Commission (CAC) standard MRL values for amoxicillin (0.5%), tetracycline (4%), and oxytetracycline (1.5%) residues in food. The tetracycline residues (130.00 to 220.35 µg/L) in pasteurized milk and sterilized milk samples (3/50; 6% each), and in processed cheese (2/50; 4%), were higher than the maximum MRL of 100 µg/L. Furthermore, the oxytetracycline residue levels (156.00 to 160.76 µg/L) in the pasteurized milk samples (3/50; 6%) were higher than the international MRL value of 100 µg/L, and the amoxicillin residue (5.57 µg/L) found in processed cheese (1/50; 2%) was also above the CAC standards’ permissible MRL limit of 4 µg/L (Figure 3A). 

**Figure 4:**
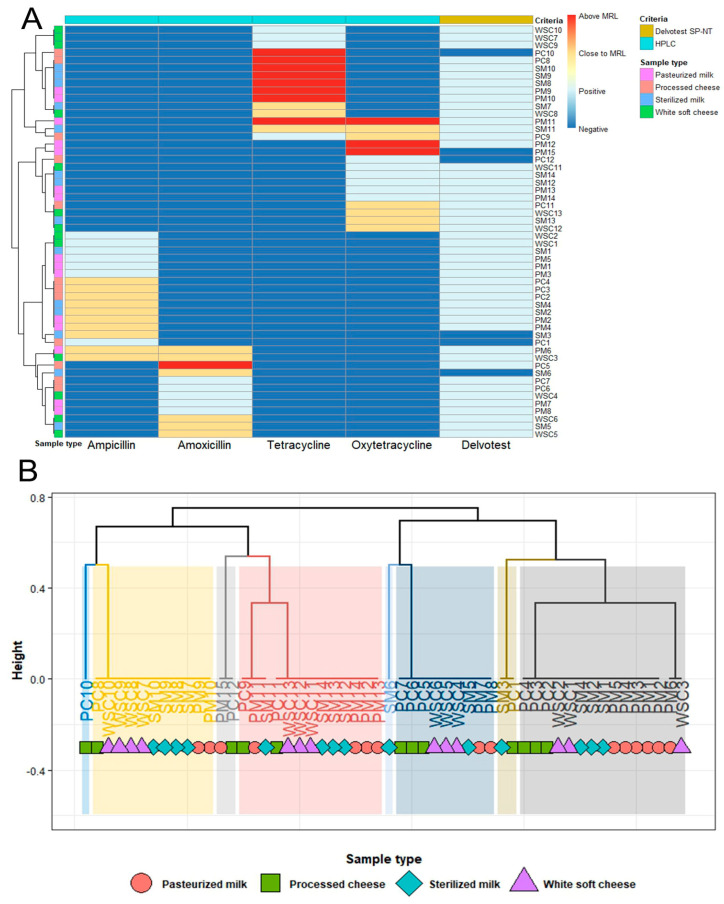
Hierarchical clustering heatmap (**A**) and hierarchical clustering dendrogram (**B**) of the positive HPLC samples based on the results of Delvotest SP-NT and HPLC concentration for various antibiotics. WSC: white soft cheese, PC: processed cheese, SM: sterilized milk, PM: pasteurized milk. The different shades of color indicate eight clusters. The values of the maximum residue limit (MRL) for ampicillin and amoxicillin = 4 µg/L each, and for tetracycline and oxytetracycline = 100 µg/L each, according to Codex Alimentarius Committee [28]. Close to MRL for ampicillin and amoxicillin > 3.5 µg/L each; for tetracycline and oxytetracycline > 85 µg/L each.

Furthermore, the antibiotic residues detected in 12.5% (25/200) of the tested samples were close to the permissible MRL limits for ampicillin (5%), amoxicillin and oxytetracycline (3% each), and tetracycline (1.5%) (Figure 3B). Ampicillin residues were found in 6% of the pasteurized milk, sterilized milk, and processed cheese samples, and in 2% of the white soft cheese, at concentrations above 3.5 µg/L (3.57–3.89 µg/L). Amoxicillin residues were detected in 6% of the white soft cheese, 4% of sterilized milk, and 2% of pasteurized milk, at concentrations (3.55–3.90 µg/L) close to the MRL of 4 µg/L. Oxytetracycline and tetracycline residues were found in 3% and 1.5% of the total samples, respectively, at concentrations above 85 µg/L (87.44–99.00 and 90.11–95.35 µg/L), which are close to the recommended MRL value of 100 µg/L. Each of the sterilized milk, white soft cheese, and processed cheese samples (4%) contained oxytetracycline residues close to the MRL value. Nevertheless, the sterilized milk (4%) and white soft cheese (2%) contained tetracycline residues close to the MRL value (Figure 3B and Figure 4A).

Hierarchical cluster analysis (HCA) (Figure 4A,B) corroborated the distinctions between the samples based on their antibiotic screening results and the concentrations of various antibiotics. The samples were distributed into eight clusters. Six clusters contained different samples.

### 2.4. Correlation Between Analytical Methods

**Figure 5:**
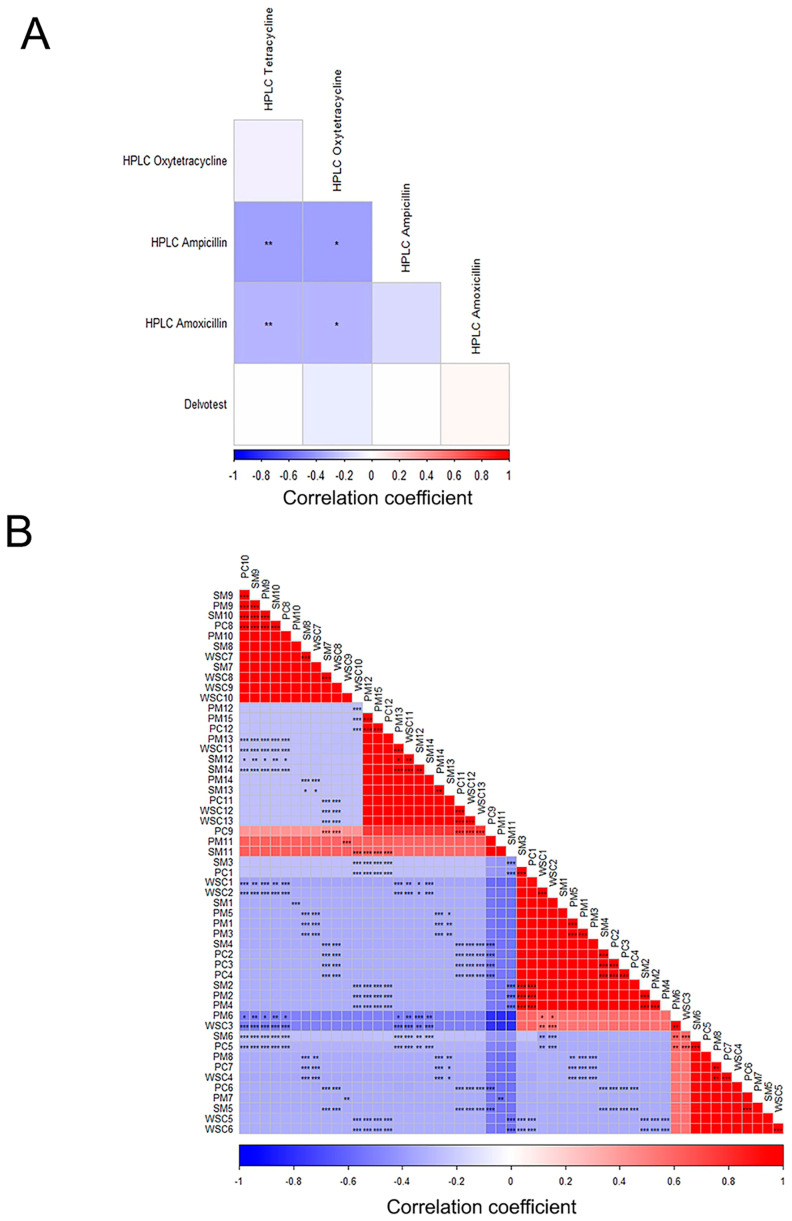
Pairwise correlation of different analyzed antibiotic residues detected by using the Delvotest SP-NT and HPLC methods (**A**) and samples (**B**). The correlation coefficients are shown as colors on the scale (positive: red and negative: blue). The more intense the color, the stronger the positive or negative correlation. Stars in correlation plots refer to significant differences: * *p* < 0.05, ** *p* < 0.01, and *** *p* < 0.001.

Pearson’s correlation of different analyzed antibiotic residues in the milk and cheese samples was performed for the comparative assessment of the results obtained by the Delvotest SP-NT and HPLC methods. A highly significant (*p* = 0.005) negative correlation was found between ampicillin and both tetracycline (r = −0.375) and oxytetracycline (r = −0.376) detection using HPLC. In addition, a significant (*p* = 0.031) negative correlation was found between amoxicillin and both tetracycline (r = −0.293) and oxytetracycline (r = −0.294). A non-significant negative correlation was observed between the Delvotest SP-NT and HPLC for the detection of ampicillin (r = −0.006), tetracycline (r = −0.004), and oxytetracycline (r = −0.075) residues in dairy products. A non-significant weak positive correlation was found between the Delvotest SP-NT and HPLC for the detection of amoxicillin (*p* = 0.779, r = 0.039) (Figure 5). 

**Figure 6:**
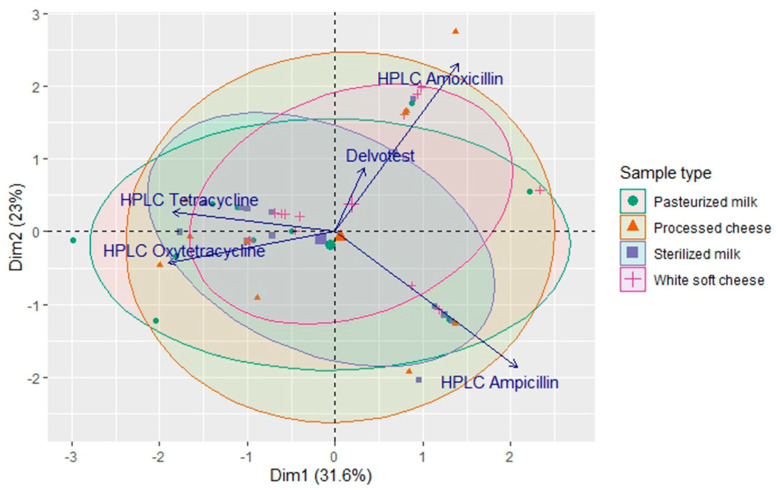
Principle component analysis (PCA) biplot of the positive samples based on the results of Delvotest SP-NT and HPLC analyses.

Overall, the principal component analysis (PCA) biplot (Figure 6) provides a visual representation of the relationships between the antibiotic residues, detection methods, and sample types. These findings suggest that the method used, and the co-occurrence of antibiotics are important factors to consider when assessing the safety of dairy products. Dim1 (31.6%) explained the greatest variation in the data. This appears to have been influenced by the differences between the sample types and the presence of certain antibiotics. Dim2 (23%) may capture additional variations related to the specific types of antibiotic residues detected. 

The biplot shows distinct clustering of the sample types. The pasteurized milk and processed cheese samples tended to cluster together, whereas the sterilized milk and white soft cheese samples clustered together. This suggests that the presence of antibiotic residues may have been influenced by the processing method used. The amoxicillin and ampicillin residues appear to be closely related, as they point in a similar direction. This may indicate that they were frequently detected in the samples. The tetracycline and oxytetracycline residues also showed a similar relationship, suggesting their co-occurrence (Figure 6). The Delvotest SP-NT test seems to be associated with a cluster of pasteurized milk and processed cheese samples, suggesting its effectiveness in these types.

### 2.5. Validation Parameters for HPLC Method 

Good linearity was observed with correlation coefficients (r) of 0.98, 0.997, 0.998, and 0.998 for ampicillin, amoxicillin, tetracycline, and oxytetracycline, respectively. The sensitivity and specificity of the HPLC method were 100%.

The accuracy of the method was determined by triplicate analyses of spiked milk samples at three fortification levels (25, 50, and 250 ng/mL). The recoveries for ampicillin, amoxicillin, tetracycline, and oxytetracycline were (100.08–102.47%), (101.12–104.9%), (109.79–102.74%), (101.17–101.71%), respectively. The lowest detectable amounts of the analyte in a sample (LOD) were 17.59, 19.31, 1, and ˂1 ng/mL for tetracycline, oxytetracycline, amoxicillin, and ampicillin, respectively. The lowest quantifiable amounts of analyte with acceptable precision and accuracy (LOQ) were 58.65, 64.36, 3, and 2 ng/mL for oxytetracycline, tetracycline, amoxicillin, and ampicillin, respectively.

### 2.6. Potential Health Risk Associated with Antibiotic Residues

To assess the potential health hazards of dietary exposure to antibiotic residues in milk and cheese, the HQ value was calculated based on the mean value of the residues in the samples. As shown in Table 3, the HQ, which compares the standard acceptable daily intake (ADI) to the estimated exposure (EDI), indicates that the overall risk associated with antibiotic residues in these dairy products is low. The HQ values were 0.041 ± 0.006 for ampicillin, 0.051 ± 0.01 for amoxicillin, 0.15 ± 0.028 for tetracycline, and 0.116 ± 0.024 for oxytetracycline in all the sample types. The EDI values were lower than the ADI values for all the tested antibiotics (Table 3). The EDI values from different sample types consumed by a 70 kg individual were 8.55 ± 1.71, 7.18 ± 1.42, 315.82 ± 59.74, and 243.84 ± 49.79 μg/day for ampicillin, amoxicillin, tetracycline, and oxytetracycline, respectively. The ADI values for antibiotics in a 70 kg individual were 210 μg/day for penicillin, 140 μg/day for amoxicillin, and 2100 μg/day for tetracycline and oxytetracycline. The *p*-values for both the EDI and the HQ calculations were highly significant (*p* < 0.0001), indicating that the differences observed between sample types and antibiotics were statistically significant.

## 3. Discussion

The alarming increase in antimicrobial-resistant bacteria worldwide poses a serious threat to global public health, akin to a silent pandemic that requires immediate action. AMR primarily spreads through the transmission of bacteria between animals and humans (zoonotic pathways) and from humans to humans (anthroponosis) within and outside healthcare settings [29]. Excessive use of antibiotics can contribute to the development of antibiotic resistance, which can directly and indirectly impact bacterial populations and hinder the treatment of infections. It is important to note that antibiotic residues in food are a major concern in this regard [30]. The presence of antibiotic residues, particularly in milk, has both economic and public health consequences. For instance, penicillin contamination in milk can hinder the fermentation process and the starter cultures used to produce cheese, buttermilk, and sour cream, leading to financial losses. Additionally, accurately identifying drug residues that can cause serious or fatal health problems in humans is crucial for ensuring public health safety [14]. Moreover, repeated exposure to low levels of antibiotics in milk can contribute to AMR crises, making infections more difficult to treat effectively [31]. A major strength of this study is the screening, quantification, and health hazard assessment of important veterinary β-lactam antibiotics and tetracycline residues in milk and cheese from Kuwait hospitals. Decision 2002/657/EC [32] mandates a combined screening and confirmatory approach for antibiotic residue control. Therefore, the selection of screening methods is crucial for the overall control process. Screening methods are pivotal to the entire control procedure [15]. Delvotest^®^ SP NT is a rapid screening test that relies on the inhibition of *Bacillus stearothermophilus* var. *calidolactis* to identify potential contaminants in milk samples. It has been validated for detecting β-lactam antibiotics such as amoxicillin, ampicillin, cefapirin, and penicillin G in raw cow milk [16], as well as aminoglycosides, macrolides, sulfonamides, tetracyclines, and diamino pyrimidine residues at levels that are at least equal to or below the MRLs, with a sensitivity of 95% [14]. Screening methods such as thin-layer chromatography and microbial inhibition tests are qualitative techniques used to detect residues. In contrast to screening methods, confirmatory methods are more accurate, specific, sensitive, expensive, time-intensive, and require trained professionals. Analytical techniques, such as Liquid Chromatography (LC) coupled with various detection modes, such as mass spectrometry (MS), UV, and HPLC, are frequently employed for quantitative confirmation [15,16,17,18]. Furthermore, immunoassay-based techniques generally have lower sensitivity for detecting LOD and LQD compared to HPLC [33,34]. The samples that tested negative in the screening test proceeded directly for further confirmation [35,36]. 

Both Delvotest SP-NT and HPLC confirmed that 48 milk and cheese samples out of 200 (24%) tested samples were positive for antibiotic residues. Additionally, six samples that were initially negative for Delvotest SP-NT were later confirmed to be positive using HPLC. Perfect agreement was observed between the Delvotest SP-NT and HPLC methods, which could provide further insight into the reliability of Delvotest SP-NT as a screening tool for practical applications in food safety testing. Nevertheless, 6.11% of the milk samples examined in Kosovo using Delvotest SP contained possible drug residues [37]. Moreover, Delvotest SP-NT had a higher false-negative rate than LC-MS/MS, as confirmed by the LC-MS/MS detection of antibiotics in milk samples collected in Algeria [38]. This could be attributed to the clear yellow and clear purple colors that are easily distinguishable for Delvotest SP-NT, whereas visually assessing samples with intermediate antimicrobial concentrations is more challenging, even for experienced technicians, making the visual interpretation of the colored reaction subjective [35,38]. Moreover, abnormal milk, such as milk from mastitis or colostrum, can interfere with the accuracy of the Delvotest^®^ SP-NT test [14].

According to the Delvotest SP-NT and HPLC results, the samples most contaminated with antibiotic residues were those obtained from pasteurized milk (30%), sterilized milk (28%), white soft cheese (26%), and processed cheese (24%). However, Rahman et al. [17] reported a lower prevalence of antibiotic residues in raw milk (7%) collected from Bangladesh, which was higher in individual samples (8%) than that in pooled samples (4%). No residues were found in the processed milk. Santamarina-García [39] found that 12.5% of samples, primarily raw milk and whey, contained antibiotics at levels close to the detection limit. Additionally, 10% of the samples tested positive for antibiotics, particularly fresh and ripened cheeses, suggesting that antibiotics accumulated during the cheesemaking process. Factors such as test efficiency, study duration, sample size, location, and prevalence of diseases in the study area can influence the variation in antibiotic residue detection rates. The use of antimicrobials in dairy cows and the subsequent presence of residues in milk are closely linked to the disease burden in the region, which may have contributed to the differing results [17]. In addition, owing to the nature of milk transportation, one cow could contaminate a bulky consignment of milk with antibiotic residues.

β-lactams and tetracyclines are the most commonly used antibiotics in food-producing animals [13]. β-lactam antibiotics continue to be a cornerstone of treatment for many patients, but their effectiveness is limited by allergic reactions and the increasing resistance of bacteria, including through enzymes called β-lactamases. Efforts to combat antibiotic resistance continue, but cross-contamination and wastewater pollution remain concerns for β-lactam antibiotics [40]. Consequently, β-lactam antibiotics have the lowest tolerance levels among all antimicrobials in the European Union (EU). EU Regulation 2377/90 sets MRLs for some β-lactam antibiotics in milk, such as penicillin G, ampicillin, and amoxicillin, at 4 μg/L [41]. 

In this sense, ampicillin was the most abundant residue in the positive samples (17/54; 31.48%) at concentrations ranging from 2.44 to 3.89 μg/L, followed by tetracycline and oxytetracycline (15/54; 27.78% each), at concentrations ranging from 54.13 to 220.3 μg/L and 41.55 to 160.7 μg/L, respectively. Amoxicillin was found in 22.22% (12/54) of the positive samples at concentrations ranging from 3.11 to 5.5 μg/L. Overall, these findings align with the observation that penicillin and tetracycline are the most commonly used veterinary antibiotics, although specific compounds and dosages may differ across farms and regions [39,42]. For instance, Ghidini et al. [41] reported a higher prevalence of penicillin G in 49.06% (26/53) of positive milk samples collected from Brescia city, in Italy, at concentrations ranging from ˂4 to 6240 ± 550 μg/L and a lower prevalence of amoxicillin (5.66%; 26/53) at concentrations ranging from 8.5 ± 0.1 to 53.7 ± 2.3 μg/L. Milk samples from Nazareth dairy farm in Ethiopia were analyzed for oxytetracycline and penicillin G residues using HPLC. Oxytetracycline (concentration range: 45–192 μg/L) and penicillin G (0–28 μg/L) were detected in 12% of the milk samples [43]. Recently, tetracycline (7.88%) and oxytetracycline (5.91%) were detected in milk samples from three Indonesian districts, with concentrations ranging from 11.7 to 49.4 ng/g for tetracycline and 11.6 to 85.6 ng/g for oxytetracycline. However, neither the oxytetracycline nor the tetracycline residues exceeded the MRL [44]. 

The issues related to antibiotic inactivation during milk processing are more critical than those encountered in other animal-based foods, primarily because milk undergoes a very short heat treatment. In addition, different antibiotics exhibit different levels of heat resistance [45,46]. Considering that pasteurization slightly reduces antibiotic residues in goat’s milk only, leaving between 70.8% and 100% of the initial concentration in the pasteurized product [27], the mean concentrations of ampicillin and amoxicillin were slightly lower in pasteurized milk (3.45 ± 0.25 μg/L) than sterilized milk, with mean concentrations of 3.54 ± 0.27 and 3.72 ± 0.17 μg/L, respectively. Nonetheless, the mean concentrations of tetracycline and oxytetracycline were higher in pasteurized milk (160.45 ± 0.24 μg/L and 120.45 ± 0.25 μg/L) than sterilized milk (130 ± 0.22 and 77.8 ± 0.28 μg/L, respectively). This agrees with the findings of a previous study showing that heat treatment of milk only partially reduces tetracycline residue concentrations and does not completely eliminate them [45]. Kellnerová et al. [47] reported that tetracycline and oxytetracycline residues in milk were only slightly reduced by pasteurization, with reduction rates of 5.74% and 15.3%, respectively. Therefore, it is important to strictly follow antibiotic withdrawal times to minimize the risk of antibiotic residues in milk [48].

To safeguard consumer health, it is essential to test milk and dairy products for antibiotic residues, as these can pose both direct and indirect health risks through the emergence of resistant bacteria and AMR development [49]. Studies have demonstrated that exposure to even low concentrations of antibiotics can be associated with a range of health problems, including obesity, cancer, reproductive issues, and teratogenic effects. However, some independent reviews suggest that the actual risk from low-dose antibiotic exposure in food may be negligible because of the limited evidence supporting a strong connection [50]. Notably, the analysis of milk and cheese samples showed that tetracycline residues exceeded the maximum MRL in 6% of both pasteurized and sterilized milk samples and in 4% of processed cheese. Additionally, the oxytetracycline levels in pasteurized milk (6%) and amoxicillin levels in processed cheese (2%) were higher than those in the permitted MRL. Ampicillin was detected in 6% of pasteurized and sterilized milk, and processed cheese samples and in 2% of white soft cheese, exceeding 3.5 µg/L. Amoxicillin was near the MRL in 6% of white soft cheese, 4% of sterilized milk, and 2% of pasteurized milk samples. The oxytetracycline and tetracycline levels approached the MRL in 3% and 1.5% of the samples, respectively. Additionally, 4% of the sterilized milk, white soft cheese, and processed cheese samples contained oxytetracycline residues close to the MRL, while the sterilized milk (4%) and white soft cheese (2%) had tetracycline residues near the MRL. This is consistent with various studies [15,51,52] that detected antibiotic residues in milk samples at levels close to or exceeding the MRL, despite efforts to comply with the MRL regulations. In addition, antibiotic concentrations below the MRLs have been reported for raw milk and cheese in Spain [39]. Antibiotic residues, even below the MRLs, can develop bacterial resistance, affect cheese microbiota, and compromise cheese quality and safety [27]. However, nearly one-third of the local fresh milk samples previously examined in Kuwait state during 2004–2005 contained levels of tested residues that exceeded the MRL, with tetracycline being the most prevalent residue detected. This evidence strongly suggests that antimicrobial drugs are being used inappropriately in livestock farming practices in Kuwait [53].

The FSM helps to determine whether the gap between the EDI and the ADI of the food hazard is wide enough to ensure safety [27]. While the overall hazard quotients are low, the potential for the development of antibiotic resistance due to long-term exposure to low levels of antibiotics should be considered. The calculated hazard quotients for ampicillin, amoxicillin, tetracycline, and oxytetracycline in milk and cheese samples were 0.041 ± 0.006, 0.051 ± 0.01, 0.15 ± 0.028, and 0.116 ± 0.024, respectively. It is worth mentioning that the EDI of the tested milk and cheese was lower than the ADI for tested antibiotics. Similarly, a previous study found that the EDI was much lower than the ADI for amoxicillin and oxytetracycline in milk and posed a low health hazard to Bangladeshi people [16]. Moreover, an Indian study found a negligible public health risk from oxytetracycline and tetracycline (hazard quotients = 0.009 and 0.004, respectively) [45]. In addition, the hazard quotients and FSM for all the samples were close to 1, indicating low health risks from consuming raw milk or whey in Spain [39].

## 4. Conclusions

The presence of one or more antibiotic residues in milk and cheese samples can potentially endanger public health. The high residue levels of ampicillin, amoxicillin, tetracycline, and oxytetracycline in milk and cheese served in Kuwait hospitals are alarming, as they may contribute to the emergence and dissemination of antimicrobial resistance. Therefore, strict regulations and enforcement are necessary to prevent excessive residue levels during dairy production. Regular monitoring of antibiotic residues in dairy products is essential for ensuring consumer safety. Adhering to MRLs for antibiotics in food-producing animals is mandatory to avoid the serious consequences of antibiotic residues. Furthermore, it is important to avoid the unnecessary use of antibiotics in food-producing animals, limit their use, and explore alternatives to antibiotics. These measures safeguard food production chains, food safety, public health, and consumer rights. By effectively managing waste milk and reducing antibiotic residues, we can significantly minimize the risks associated with antimicrobial resistance and improve the health and productivity of livestock. Hence, future research on developing innovative methods to eliminate antibiotics from milk and milk products without compromising their quality is warranted. Additionally, investigating the factors that influence antibiotic degradation or persistence during milk production could provide valuable insights.

## 5. Materials and Methods

### 5.1. Sampling 

To evaluate the frequency and concentration of antimicrobial residues in milk and its products and their adherence to food safety regulations, 200 milk and milk product samples, including pasteurized milk, sterile milk, soft white cheese, and processed cheese (50 of each), were collected from January to May 2024 from different governmental hospitals in the State of Kuwait. Milk and milk products were sourced locally. The samples were transported in an icebox to the laboratory and stored at −80 °C for subsequent analysis. The cheese samples were thawed at 4 °C for 24 h and then brought to room temperature for an hour before testing. Ten grams of cheese were mixed with 20 g of trisodium citrate (20%, *w*/*w*; Merck KGaA, Darmstadt, Germany) in a stomacher bag and blended twice for three minutes at 40 °C. The mixture was then centrifuged at 9000× *g* for 10 min and the supernatant was collected for analysis [27]. These samples were processed using a screening test (microbial inhibitor test: Delvotest^®^ SP NT) and confirmed by HPLC analysis for quantification of β-lactam and tetracycline residues.

### 5.2. Chemicals and Reagents

The chemicals used in the experiments were purchased from reputable suppliers. Sodium acetate, calcium chloride, sodium ethylenediaminetetraacetic acid (EDTA), disodium hydrogen phosphate, citric acid, and sodium hydroxide were obtained from Sigma (Burbank, CA, USA). Hydrochloric acid, ammonium hydroxide, phosphoric acid, trichloroacetic acid, HPLC-grade methanol, and acetonitrile were purchased from Merck (Darmstadt, Germany). HPLC-grade water was purified using a Milli-Q system (Merck Millipore, Burlington, MA, USA). Mcllvaine buffer (0.01 M; pH 4) was prepared from 11.8 g citric acid monohydrate, 13.72 g disodium hydrogen phosphate dihydrate, and 33.62 g ethylenediaminetetraacetic acid disodium salt in 1 L water [54]. Analytical-grade antibiotics (commercial reference), including amoxicillin (A8523), ampicillin (A9393), oxytetracycline (O4636), and tetracycline (T3258), were purchased from Sigma (St. Louis, MO, USA).

### 5.3. Screening Method Analysis

Residue testing was screened on the day of sample collection and results were recorded as “positive” or “negative” for presence of the antibiotic residue. Delvotest^®^ SP NT (from DSM Food Specialties BV, Delft, Nederland) utilizes ampoules containing solid agar medium, *Bacillus stearothermophilus* var. *calidolactis* (highly sensitive to most antibiotics), and bromocresol purple as a pH indicator. Each ampoule was clearly identified with a sample number, and then 0.1 mL of sample were added, by using a disposable pipette, to the identified ampoule after perforation of the aluminum foil covering the ampoule. A new pipette was used for each sample to avoid contamination of the samples by each other. The ampoules were incubated at 64 °C (±2 °C) for 3 h in a preheated lidded water bath. A β-lactam-positive control (4 µg kg^−1^ ampicillin), a tetracycline-positive control (1000 µg kg^–1^ oxytetracycline), and negative control were included in the test to ensure the reliability of the kit. The results were assessed immediately following incubation according to the manufacturer’s instructions. The development of yellow color indicated the absence of the antibiotic residues in the sample; therefore, the active metabolism of *B. stearothermophilus* var. *calidolactis* changed the bromocresol purple indicator to yellow and indicated a negative result. The persistence of the purple color of the medium indicated a positive result for the presence of antibiotic residues in the sample (which inhibits *B. stearothermophilus* var. *calidolactis*).

### 5.4. HPLC Analysis for Quantitative Detection of β-Lactam and Tetracycline Residues

The samples were further analyzed using HPLC to determine the quantity of four commonly used veterinary antimicrobials: penicillin, amoxicillin, tetracycline, and oxytetracycline. 

#### 5.4.1. Preparation of Samples for Quantification of β-Lactam Antibiotic Residues

In total, 5 ml of each sample were mixed with 400 µL of 10% aqueous solution of acetic acid (Merck KGaA, Darmstadt, Germany) in a sterilized centrifuge tube. The solution was vortexed (IKA vortex Genius 3, Fisher Scientific, Uk Ltd., Loughborough, UK) for one minute, and then centrifuged at 3500 rpm for 10 min at 4 °C (Centurion, Washington, DC, USA). The clear supernatant was taken carefully and filtered using a 13 mm/0.45 µm nylon syringe filter (Thermo Fisher Scientific Inc., Waltham, MA, USA), and the filtrate was taken into a 2 mL autosampler HPLC-vial (Merck KGaA, Darmstadt, Germany). CI8 SPE cartridges were attached to the vacuum manifold and preconditioned with 4 mL methanol, and then conditioned with 1 mL 0.005 M potassium dihydrogen phosphate (pH 6.8). The collected supernatant was transferred onto the cartridge and the flow rate adjusted to 1–2 mL/min. After filtering, the cartridge was washed with 1 mL of water and dried for 20 min. In total, 1 ml of elution solution (60 water: 40 methanol) was applied, and then the elute was collected into a clean container and evaporated under steam of nitrogen (TurboVap LV evaporator, Aliper Life Sciences, Hopkinton, MA, USA) at 50 °C. The residue resuspended in mobile phase, filtered, and stored in autosampler vials for further analysis. Twenty microliters of filtrate were injected into the HPLC system [41]. 

#### 5.4.2. Samples Preparation for Quantification of Tetracyclines Residues

In total, 5 ml of each sample were mixed with 2 mL of 20% trichloroacetic acid (TCA, Merck, Darmstadt, Germany) solution into a polypropylene centrifugal tube. The solution was vortexed for 5 min, and 20 mL of Mcllvaine buffer were added. Next, the mixture was centrifuged at 7500 rpm for 15 min at 4 °C. The supernatant was filtered through Whatman™ filter paper, collected in a clean beaker, and underwent further purification steps using a SPE HLB C18 cartridge preconditioned with 5 mL of methanol and 3 mL of water. The cartridge was then washed with 3 mL of 3% methanol in water, and tetracyclines were eluted with 5 mL of methanol. The eluted substance was collected in a clean container and evaporated to dryness under vacuum at a temperature of 35 °C. The residue was reconstituted in 3 mL of methanol, filtered using a 0.45 µm nylon syringe filter, and stored in an autosampler vial for further analysis. Twenty microliters of the sample were injected into the HPLC system [33].

#### 5.4.3. Chromatographic Analysis of Antibiotic Residues

Mobile phase composition for β-lactam antibiotic residues, as previously described [41], was water and acetonitrile, both acidified with 0.1% formic acid at 1 mL min^−1^ constant flow from 100% water to 100% acetonitrile in 6 min and from 100% acetonitrile to 100% water from 6 to 12 min. 

An aqueous phase of 0.075 mol/L sodium acetate, 0.035 mol/L calcium chloride, and 0.025 mol/L sodium EDTA, pH 7.0, and an organic phase of methanol: acetonitrile (75:25, *v*/*v*) was used in tetracycline residues according to Mamani et al. [55]. Gradient elution of 90:10 aqueous phase: organic phase *v*/*v* to 50:50 aqueous phase: organic phase *v*/*v* (0–30 min), and then to 90:10 aqueous phase: organic phase *v*/*v* (30–35 min) was used. 

The separation of antimicrobials was performed on a Hypersil Eclipse XDP C18 (5 µm, 250 mm × 4.6 mm). The flow rate was adjusted at 0.8 mL/min at 27 °C. Quantitative measurements were carried out at 210 nm (ampicillin and amoxicillin) and 355 nm (tetracycline and oxytetracycline).

HPLC measurements were carried out using Agilent series 1200 quaternary gradient chromatographic system (Agilent Technologies, Madrid, Spain), coupled to multi-wave detector. Data acquisitions were performed by Chemstation Agilent Chromatography Software (version B.04.03). The separation of the antibiotics by HPLC was performed on a reversed-phase Agilent C18 column (250 mm, 4.6 mm internal diameter, 5 μm; Thermo Scientific, USA). The solid phase extraction was performed using Agilent Bond Elut C18-SPE cartridges (500 mg, 6 mL) connected to a 20-port solid phase extractor that was connected to an SPE vacuum manifold (Merck, Germany) for suction of samples.

#### 5.4.4. Preparation of Standards 

Stock solutions of amoxicillin and ampicillin (100 µg/mL) were prepared in deionized water and filtered with a 0.45 µm syringe. Stock standard solutions of oxytetracycline and tetracycline (1 mg/mL) were prepared by dissolving 10 mg of each in 10 mL of methanol. The stock solutions were kept in dark bottles at 4 °C for two weeks, and the working solutions were freshly prepared in HPLC-grade water. Fresh milk free of antimicrobial residues was spiked with varying levels of a standard solution to produce different test concentrations. The working solutions of amoxicillin and ampicillin at concentrations of 0.01, 0.025, 0.05, 0.1, 0.2, 0.5, 1, 2, 5, and 10 µg/mL and tetracycline and oxytetracycline at concentrations of 0.005, 0.01, 0.02, 0.05, 0.1, 0.2, 0.5, 1, 2.1, and 4.2 µg/mL were used for sample spiking for preparation of calibration curves. 

#### 5.4.5. HPLC Validation

The validation was performed using spiked milk samples, following the guidelines set by the European Commission’s Decision 2002/657/EC [32]. 

Linearity, sensitivity, specificity, accuracy, limit of detection (LOD), and limit of quantitation (LOQ) were the determined criteria. The linearity of the response was evaluated by analyzing milk samples fortified with ampicillin, amoxicillin, tetracycline, and oxytetracycline at five different concentrations (25, 50, 100, 200, and 250 ng/mL). Each concentration was analyzed in triplicate. The standard calibration curves were constructed by plotting concentrations against peak area ratio of analyte to standard. 

From the standard deviation (σ) of y-intercepts of regression analysis and the slope of the calibration curve (m), LOD was calculated using the equation 3 σ/m and LOQ was calculated through 10 σ/m. 

The sensitivity of the method, which is the change in analytical signal units per nanogram of analyte, was represented by the slope of the calibration curve. The specificity was evaluated by analyzing ten different blank milk samples to check whether any naturally occurring substances in the milk interfered with the analysis. The accuracy, expressed as the percentage of recovery, was determined by analyzing spiked milk samples at three different concentrations (25, 50, and 250 ng/mL). Each concentration was analyzed in triplicate. The recoveries were calculated by comparing the peak area of the measured concentration to the peak area of the spiked concentration. 

### 5.5. Determination of the Potential Risk Associated with the Antibiotic Residues 

The FSM was calculated considering HQ and EDI, as previously described [17,27], to evaluate the potential health hazards associated with consuming antibiotic-contaminated dairy products. FSM and HQ below or equal to one means there is little risk, while a higher value suggests potential harm. However, these values do not give the exact probability of this harm happening.

Hazard quotient = Estimated daily intake/accepted daily intake [17,56]

EDI = (concentration of residue as μg/kg) × (daily intake of food in kg/person)/Adult body weight (70 kg).

Acceptable daily intakes (ADIs) of tetracycline, oxytetracycline, ampicillin, and amoxicillin were derived from the Codex Alimentarius Commission (CAC) standards [28]. The ADIs considered for tetracycline and oxytetracycline were 30 μg/kg of body weight, ampicillin 3 μg/kg of body weight, and 2 μg/kg of body weight for amoxicillin.

Calculated daily intake from dairy product consumption was 250 mL milk per day and 60 g cheese per day [57,58].

The values of the MRL for ampicillin and amoxicillin = 4 µg/L each and tetracycline and oxytetracycline = 100 µg/L each, according to CAC [28].

### 5.6. Data Analysis

The data were analyzed using SPSS version 26 (IBM Corp., Armonk, NY, USA). Chi-squared test was used to compare categorical variables, and a one-way ANOVA was used to compare numerical variables. Percentages and frequencies were used for describing categorical variables, while means and standard error of the mean (SEM) were calculated for the continuous variables. To confirm the normal distribution of the data, the Shapiro–Wilk test and Q-Q plots were used. Moreover, Cohen’s kappa coefficient and Pearson correlation were used to evaluate the agreements and detect the association between Delvotest SP-NT and HPLC, respectively. A *p*-value of 0.05 was used as a cutoff level for significance. All graphs were generated by R-software version 4.3.1 (https://www.r-project.org/ accessed on 4 August 2024).

## Figures and Tables

**Table 1:** Statistical agreement between results obtained by Delvotest SP-NT and HPLC.

		HPLC		
		HPLC Non-Conform	HPLC Conform	Total
Delvotest SP-NT	Delvotest negative	146	6	152
	Delvotest positive	0	48	48
	Total	146	54	200

Total numbers for each results category were compared and Cohen’s kappa was calculated (Cohen’s kappa = 0.921, *p* < 0.0001).

**Table 2:** Comparison of the positive results obtained by HPLC and Delvotest SP-NT given as frequency (%) of the measurement appearances.

Antibiotic	Sample Type	No. of Results, Frequency (%)	
		HPLC Conform and Delvotest Negative	HPLC Conform and Delvotest Positive
Ampicillin	Pasteurized milk	0	6 (12)
	Processed cheese	1 (2)	3 (6)
	Sterilized milk	1 (2)	3 (6)
	White soft cheese	0	3 (6)
	Total	2 (1)	15 (7.5)
Amoxicillin	Pasteurized milk	0	3 (6)
	Processed cheese	0	3 (6)
	Sterilized milk	1 (2)	1 (2)
	White soft cheese	0	4 (8)
	Total	1 (0.5)	11 (5.5)
Tetracycline	Pasteurized milk	0	3 (6)
	Processed cheese	1 (2)	2 (4)
	Sterilized milk	0	5 (10)
	White soft cheese	0	4 (8)
	Total	1 (0.5)	14 (7)
Oxytetracycline	Pasteurized milk	1 (2)	4 (8)
	Processed cheese	1 (2)	2 (2)
	Sterilized milk	0	4 (8)
	White soft cheese	0	3 (6)
	Total	2 (1)	13 (6.5)

**Table 3:** Estimation of risk assessment by hazard quotient for mean concentrations of residues in milk and milk product samples.

Antibiotic Tested	ADI ^a^ (μg/Day)	Sample Type	EDI ^b^ (μg/Day)	Hazard Quotient
Ampicillin	210	Pasteurized Milk	12.32 ± 0.399 ^a^	0.059 ± 0.002 ^a^
		Sterilized Milk	12.57 ± 0.41 ^a^	0.059 ± 0.002 ^a^
		White soft cheese	2.77 ± 0.364 ^b^	0.013 ± 0.002 ^b^
		Processed cheese	3.19 ± 0.14 ^b^	0.015 ± 0.001 ^b^
		*p*-value	<0.0001	<0.0001
		Total	8.55 ± 1.71	0.041 ± 0.006
Amoxicillin	140	Pasteurized Milk	12.32 ± 0.515 ^a^	0.088 ± 0.004 ^a^
		Sterilized Milk	13.27 ± 0.589 ^a^	0.095 ± 0.004 ^a^
		White soft cheese	3.13 ± 0.16 ^b^	0.022 ± 0.001 ^b^
		Processed cheese	3.38 ± 0.667 ^b^	0.024 ± 0.005 ^b^
		*p*-value	<0.0001	<0.0001
		Total	7.18 ± 1.42	0.051 ± 0.01
Tetracycline	2100	Pasteurized Milk	573.09 ± 38.09 ^a^	0.273 ± 0.018 ^a^
		Sterilized Milk	464.71 ± 63.22 ^a^	0.221 ± 0.03 ^a^
		White soft cheese	64.38 ± 6.55 ^b^	0.031 ± 0.003 ^b^
		Processed cheese	145.63 ± 43.19 ^b^	0.069 ± 0.021 ^b^
		*p*-value	<0.0001	<0.0001
		Total	315.82 ± 59.74	0.15 ± 0.028
Oxytetracycline	2100	Pasteurized Milk	430.29 ± 84.17 ^a^	0.205 ± 0.04 ^a^
		Sterilized Milk	277.86 ± 26.52 ^ab^	0.132 ± 0.013 ^ab^
		White soft cheese	61.99 ± 13.19 ^b^	0.029 ± 0.006 ^b^
		Processed cheese	69.59 ± 11.67 ^b^	0.033 ± 0.006 ^b^
		*p*-value	0.003	0.003
		Total	243.84 ± 49.79	0.116 ± 0.024

bw: Body weight. ^a^ ADI: Acceptable daily intake data for tetracycline, oxytetracycline, ampicillin, and amoxicillin (μg/kg bw/day) were derived from the Codex Alimentarius Commission standards [28] and calculated for 70 kg body weight individuals. ^b^ EDI: Estimated daily intake (μg/kg bw/day) was calculated using the following formula: (milk consumption × mean concentration of residue in milk)/body weight (70 kg) [17].

## Data Availability

Data are contained within the article or Supplementary Material.

## Supplementary Materials

The following supporting information can be downloaded at: https://www.mdpi.com/article/10.3390/antibiotics13111073/s1. Table S1: Prevalence of antibiotic residues in milk and milk product samples using Delvotest SP-NT and HPLC analysis; Table S2: Concentrations of different antibiotic residues in milk and cheese samples using HPLC.
