# Supplementary material for: Antibiotic Residues in Milk and Milk-Based Products Served in Kuwait Hospitals: Multi-Hazard Risk Assessment
Source: Antibiotics (Basel). 2024 Nov 11;13(11):1073. doi: 10.3390/antibiotics13111073 (PMC11591502; doi:10.3390/antibiotics13111073)
Supplement: Supplementary file 1 [file antibiotics-13-01073-s001.zip › antibiotics-3249364-supplementary.pdf]

Supplementary Table S1: Prevalence of antibiotic residues in milk and milk product samples using Delvotest SP-NT and HPLC analysis

	Delvotest SP-NT		Antibiotic tested by HPLC (N, %)				HPLC	
**Type of samples (N)**	No. of positive samples	% of positive (95 % CI)	Ampicillin	Amoxicillin	Tetracycline	Oxytetracycline	Overall positive	Overall percentage (95 % CI)
Pasteurized milk (50)	14	28 (15-41)	6 (12)	3 (6)	3 (6)	5 (10)	15[a]	30 (17-43) [a]
Sterilized milk (50)	12	24 (12-36)	4 (8)	2 (4)	5 (10)	4 (8)	14[b]	28 (15-41) [b]
White soft cheese (50)	13	26 (13-39)	3 (6)	4 (8)	4 (8)	3 (6)	13[c]	26 (13-39) [c]
Processed cheese (50)	9	18 (7-29)	4 (8)	3 (6)	3 (6)	3 (6)	12[d]	24 (12-36) [d]
***p*-value**	**0.73**		**0.811**	**0.976**	**0.935**	**0.935**		**0.95**
Total samples (200)	48	24 (18-30)	17 (8.5)	12 (6)	15 (7.5)	15 (7.5)	54[e]	27 (21-33) [e]

[a]Two samples were contaminated with two groups of antibiotics (Ampicillin & Amoxicillin, and Tetracycline & Oxytetracycline).
[b]One sample was contaminated with two groups of antibiotics (Tetracycline and Oxytetracycline).
[c]One sample was contaminated with two groups of antibiotics ([b]One sample was contaminated with two groups of antibiotics (Tetracycline and Oxytetracycline).
[d]One sample was contaminated with two groups of antibiotics (Tetracycline and Oxytetracycline).
[e]Five samples were contaminated with more than one group of antibiotics.

Supplementary Table S2: Concentration of different antibiotic residues in milk and cheese samples using HPLC

Antibiotic tested	Sample type	No. of positive samples (%)	Minimum concentration (µg/l)	Maximum concentration (µg/l)	Mean ± SEM (µg/l)
**Ampicillin**	Pasteurized Milk	6 (12)	3.20	3.70	3.45 ± 0.25
	Sterilized Milk	4 (8)	3.22	3.78	3.54 ± 0.27
	White soft cheese	3 (6)	2.44	3.34	3.45 ± 0.23
	Processed cheese	4 (8)	3.21	3.89	3.89 ± 0.28
	***p*-value**				0.738
	**Total samples**	**17 (8.5)**	**2.44**	**3.89**	**3.492 ± 0.094**
**Amoxicillin**	Pasteurized Milk	3 (6)	3.20	3.70	3.45 ± 0.25
	Sterilized Milk	2 (4)	3.55	3.88	3.72 ± 0.17
	White soft cheese	4 (8)	3.11	3.90	3.65 ± 0.24
	Processed cheese	3 (6)	3.13	5.5	3.95 ± 0.15
	***p*-value**				0.88
	**Total samples**	**12 (6)**	**3.11**	**5.5**	**3.685 ± 0.186**
**Tetracycline**	Pasteurized Milk	3 (6)	144.5	180.70	160.45 ± 0.24
	Sterilized Milk	5 (10)	95.3	191.3	130 ± 0.22
	White soft cheese	4 (8)	54.13	90.11	75.12 ± 0.31
	Processed cheese	3 (6)	69.12	220.3	170.3 ± 0.27
	***p*-value**				0.828
	**Total samples**	**15 (7.5)**	**54.13**	**220.3**	**129.477 ± 14.22**
**Oxytetracycline**	Pasteurized Milk	5 (10)	45.5	160.70	120.45 ± 0.25
	Sterilized Milk	4 (8)	65.3	91.3	77.8 ± 0.28
	White soft cheese	3 (6)	41.55	87.44	72.23 ± 0.21
	Processed cheese	3 (6)	54.44	90.12	82.4 ± 0.27
	***p*-value**				0.438
	**Total samples**	**15 (7.5)**	**41.55**	**160.7**	**91.86 ± 9.92**
